# Limited Ferromagnetic Interactions in Monolayers of MPS_3_ (M = Mn and Ni)

**DOI:** 10.1021/acs.jpcc.2c00646

**Published:** 2022-04-12

**Authors:** Carmine Autieri, Giuseppe Cuono, Canio Noce, Milosz Rybak, Kamila M. Kotur, Cliò Efthimia Agrapidis, Krzysztof Wohlfeld, Magdalena Birowska

**Affiliations:** † International Research Centre Magtop, Institute of Physics, Polish Academy of Sciences, Aleja Lotników 32/46, PL-02668 Warsaw, Poland; ‡ Dipartimento di Fisica “E.R. Caianiello”, Università degli Studi di Salerno, I-84084 Fisciano, Salerno, Italy; § Department of Semiconductor Materials Engineering, Faculty of Fundamental Problems of Technology, Wrocław University of Science and Technology, Wybrzeże Wyspiańskiego 27, PL-50370 Wrocław, Poland; ∥ Faculty of Physics, University of Warsaw, Pasteura 5, PL-02093 Warsaw, Poland; ⊥ Consiglio Nazionale delle Ricerche CNR-SPIN, UOS Salerno, I-84084 Fisciano, Salerno, Italy

## Abstract

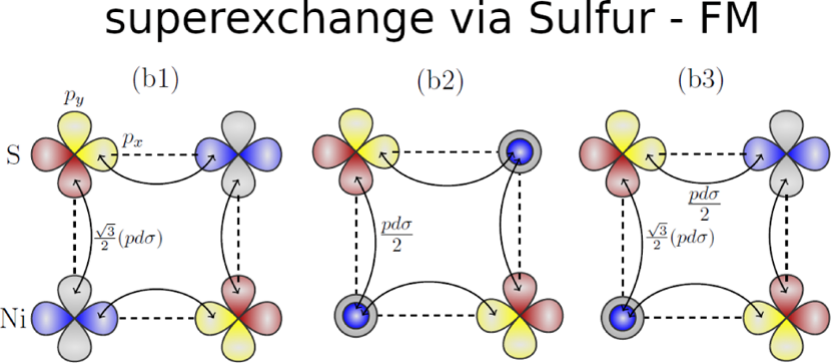

We present a systematic study of the electronic and magnetic properties of two-dimensional ordered alloys, consisting of two representative hosts (MnPS_3_ and NiPS_3_) of transition metal phosphorus trichalcogenides doped with 3d elements. For both hosts, our DFT + *U* calculations are able to qualitatively reproduce the ratios and signs of all experimentally observed magnetic couplings. The relative strength of all antiferromagnetic exchange couplings, both in MnPS_3_ and in NiPS_3_, can successfully be explained using an effective direct exchange model: it reveals that the third-neighbor exchange dominates in NiPS_3_ due to the filling of the t_2g_ subshell, whereas for MnPS_3_, the first-neighbor exchange prevails, owing to the presence of the t_2g_ magnetism. On the other hand, the nearest neighbor ferromagnetic coupling in NiPS_3_ can only be explained using a more complex superexchange model and is (also) largely triggered by the absence of the t_2g_ magnetism. For the doped systems, the DFT + *U* calculations revealed that magnetic impurities do not affect the magnetic ordering observed in the pure phases, and thus, in general in these systems, ferromagnetism may not be easily induced by such a kind of elemental doping. However, unlike for the hosts, the first and second (dopant–host) exchange couplings are of similar order of magnitude. This leads to frustration in the case of antiferromagnetic coupling and may be one of the reasons of the observed lower magnetic ordering temperature of the doped systems.

## Introduction

Nonmagnetic van der Waals layered materials such as transition metal dichalcogenides have been extensively studied over the last several years.^1^ Just recently, the intrinsic ferromagnetism in the true 2D limit has been reported,^2^ initiating increasing excitement in the spintronics and 2D material communities. In particular, the long-range magnetic order has been observed for insulating CrI_3_,^3^ semiconducting Cr_2_Ge_2_Te_6_,^4^ and metallic Fe_3_GeTe_2_^5^ compounds. In addition, topological spin structures have been predicted for 2D materials.^6^

Currently, the attention is on transition metal phosphorus trichalcogenides (MPX_3_, where M stands for a transition atom and X = S and Se), which could be easily exfoliated down to monolayers^7,8^ and are semiconducting materials with a wide range of band gaps.^9^ The MPX_3_ structures exhibit various antiferromagnetic (AFM) arrangements within the magnetic ions, which are theoretically expected to be measurable using different light polarizations.^10^ Interestingly, the metal to insulator transition and superconductivity phase have been observed in this compound family.^11−13^ In particular, the AFM insulator phase in bulk FePS_3_ can be melted and transformed into the superconducting phase under high pressure, providing similarity to the high-*T*_c_ cuprate phase diagram.^14^ In addition, theoretical predictions point to the existence of a large binding energy of excitons in MnPS_3_, whereas the experimental reports have observed excitons in few layers of NiPS_3_ strongly related to magnetic order.^13,15,16^ Recent reports have demonstrated an all-optical control of the magnetic anisotropy in NiPS_3_ by tuning the photon energy in resonance with an orbital transition between crystal field-split levels.^17^ The aforementioned demonstrates that this family of compounds is an ideal platform to study correlation effects in the true 2D limit.

In contrast to ferromagnetic (FM) materials, the antiferromagnets exhibit limited applications, mostly in the terahertz regime as ultra-fast components or specialized embedded memory–logic devices.^18−20^ Most of the current applications of magnetic crystals are based on the FM semiconductors, for which the band gap and FM order are crucial factors. The magnetic-phase transitions for the MPX_3_ materials can be accomplished by applying stress,^21^ changing the carrier concentration or applying voltage.^22^ In addition, the “M” atoms in MPX_3_ crystals might be substituted with other transition metal atoms inducing the FM order, as recently reported for a particular concentration of the non-magnetic dopants in the CrPSe_3_ host, resulting in a half-metallic FM state.^23^ Moreover, a series of mixed systems in a bulk form^24−35^ has been experimentally realized, thus entering a new playground of magnetic phases.

In addition, the magnetic and electronic properties of MPX_3_ materials and critical Néel temperature (from *T*_N_ = 78 K for MnPS_3_^36−38^ up to *T*_N_ = 155 K for NiPS_3_^36,37^) strongly depend on the type of magnetic ion in the host. Thus, an elemental substitution could be an efficient way to tune the magnetic properties of atomically thin layers, by changing the lattice parameters and magnetic moments. These quantities result in manipulation of the exchange interactions that could be an effective way to engineer highly functional materials, similar to magnetic heterostructures.^39^ In particular, the main reason behind the idea that adding ions with the partially filled d-shells into the system might lead to the enhancement of the FM interactions is related to the so-called double-exchange mechanism:^40^ the hopping between two correlated ions with different valences (and thus comparable Coulomb interactions) and relatively strong Hund’s exchange is energetically favored provided that the spins on the neighboring ions are aligned in a parallel fashion. Such a mechanism might for instance be at play along the Cr–Mn bond: here the hopping from the manganese e_g_ orbitals (e_g_^2^ configuration) to the chromium e_g_ orbitals (e_g_^1^ configuration) lowers the total energy of the system provided that the spins on Cr and Mn are aligned ferromagnetically (for the antiparallel configuration, the strong Hund’s exchange makes such a hopping energetically unfavorable). Note that such a double-exchange mechanism is similar to the one observed in the doped manganites which are FM—albeit here it is a bit more complex, for it involves two different magnetic ions (nevertheless, the latter should not be that important, for Cr and Mn have comparable values of the Coulomb interactions, *cf.* Table II of ref (41)).

Altogether, in this work, we study the magnetic properties of the MPX_3_ monolayers and try to understand (i) how FM interactions can be stabilized in these compounds and (ii) whether one can easily modify these compounds by elemental doping so that the FM interactions can be strengthened. To this end, we examine two representative hosts MnPS_3_ and NiPS_3_ in the undoped case and at a particular doping concentration of magnetic ions. Using first-principles calculations, we give qualitative and quantitative explanations of the origin of the exchange coupling strengths up to the third nearest neighbors and their respective ratios for MnPS_3_ and NiPS_3_ structures. Considering the model Hamiltonians with *ab initio* parametrization, we discuss the competition between the direct exchange and the superexchange mechanisms for the host structures. Next, we study various substantial sites of dopant atoms with mixed spins and mixed nearest-neighbor magnetic interactions. Here, again using first-principles calculations, we examine in detail the mixed exchange coupling parameters between the metal host and dopant atoms.

In the next section, we describe the computational details. In the third section, we will present our Results and Discussion while the last section is devoted to the Conclusions.

## Methods

### DFT Computational Details

The first-principles calculations are performed in the framework of spin-polarized density functional theory (DFT) as is implemented in the VASP code.^42,43^ The electron–ion interaction is modeled by using PAW pseudopotentials^44,45^ with 3s and 3p states for P and S atoms and 3d and 4s states for Mn, Ni, and Cr being treated as valence states. The Perdew–Burke–Ernzerhof (PBE) exchange–correlation functional is employed.^46^ The kinetic energy cutoff for the plane-wave expansion of the wave functions is set to 400 eV. A *k*-mesh of 10 × 6 × 2 is taken to sample an irreducible first Brillouin zone of the rectangular planar cell (see Figure S1 in Supporting Information) containing 20 atoms including four transition metal atoms. The lattice parameters have been fully optimized within the PBE + *U* approach for the magnetic ground state of the monolayers, assuming the rectangular supercell. In particular, the magnetic ground state (Hubbard *U* parameter) for MnPS_3_ and NiPS_3_ is AFM-N (*U* = 5 eV) and AFM-z (*U* = 6 eV), respectively. In the case of (2 × 2) supercell, which consists of four primitive hexagonal unit cells, the 5 × 5 × 1 *k*-mesh is chosen to obtain the optimized position of the atoms. Considering density of states (DOS) calculations, the denser *k*-mesh equal to 10 × 10 × 2 *k*-points is taken into account. The convergence criteria for the energy and force are set to 10^–5^ eV and 10^–3^ eV/Å, respectively. In order to properly model a monolayer system, 20 Å of a vacuum is added to neglect the spurious interaction between the image cells. Note that the standard exchange–correlation functionals are insufficient to account for a non-local nature of dispersive forces, which are crucial for layered materials and adsorption molecules on the surfaces.^47−50^ Thus, the semi-empirical Grimme method^51^ with a D3 parametrization is applied.^52^ For the 2D materials, we can use the HSE for a better description of the gap;^53^ however, in the case of magnetism also, GGA + *U* provides the same effect. We employ the GGA + *U* formalism proposed by Dudarev^54^ to properly account for on-site Coulomb repulsion between 3d electrons of transition metal ions, by using effective Hubbard *U* parameters.

Note that the proper choice of the *U* values is not straightforward due to the lack of accurate experimental information on electronic properties. Also, the common choice to compare the band gaps obtained in DFT with experiments to judge the *U* values also need caution due to the fact that one-particle Kohn–Sham DOS cannot be directly compared to the measured data. Thus, we decided to compute the Hubbard *U* using the linear response method proposed by Cococcioni^55^ for the monolayers of MnPS_3_ and NiPS_3_, and we obtained 5.6 eV for the Ni and 5.3 eV for the Mn. Therefore, we have used *U*_Cr_ = 4 eV, *U*_Mn_ = 5 eV, and *U*_Ni_ = 6 eV similar to the linear response results, which are typical *U* values reported for MPX_3_ materials in previous reports.^21^ Moreover, our Coulombic repulsions are close to the typical values of *U* in semiconductor compounds. Indeed, we find *U* = 6.4 for Ni^2+^ and *U* = 3.5 eV for Cr in oxides,^56^ while the typical value of *U* for Mn^2+^ is *U* = 5 eV.^57,58^

To calculate the AFM direct exchange for MnPS_3_ and NiPS_3_, we extracted the real-space tight-binding Hamiltonian with atom-centered Wannier functions with d-like orbital projections on the transition metals using the Wannier90 code.^59,60^ We calculated separately the hopping parameters for the orbitals, symmetric and antisymmetric with respect to the basal plane. The different symmetry and the separation in energy help to disentangle the two subsectors of the d manifold.^61,62^ The calculation of the hopping parameters was carried out in the non-magnetic case to get rid of the magnetic effects and evaluate just the bare-band structure hopping parameters, and then, the hopping parameters will be used for the model Hamiltonian part.^63^ In order to have parameters to use for the model Hamiltonian, we do not perform the maximum localization so as to have the Wannier function basis of our tight-biding model as close as possible to the atomic orbitals.

## Results and Discussion

The results are presented as following: first, we present the results for the magnetic ground state of the hosts (pure MnPS_3_ and NiPS_3_ systems). In particular, we consider the exchange couplings within the DFT + *U* approach. Next, the AFM exchange mechanism is discussed within the minimal many-body model, and the AFM exchange coupling strengths are evaluated numerically using the Wannier basis with *ab initio* parametrization. Next, the qualitative explanation of the FM superexchange is presented. Finally, we present comprehensive studies of electronic and magnetic properties of benchmark alloys with a fixed concentration of dopants. The elemental substitution is employed at various atomic sites of the honeycomb lattice.

### Undoped Hosts MnPS_3_ and NiPS_3_

#### Magnetic Couplings within the DFT + *U* Approach

**Figure 1:**
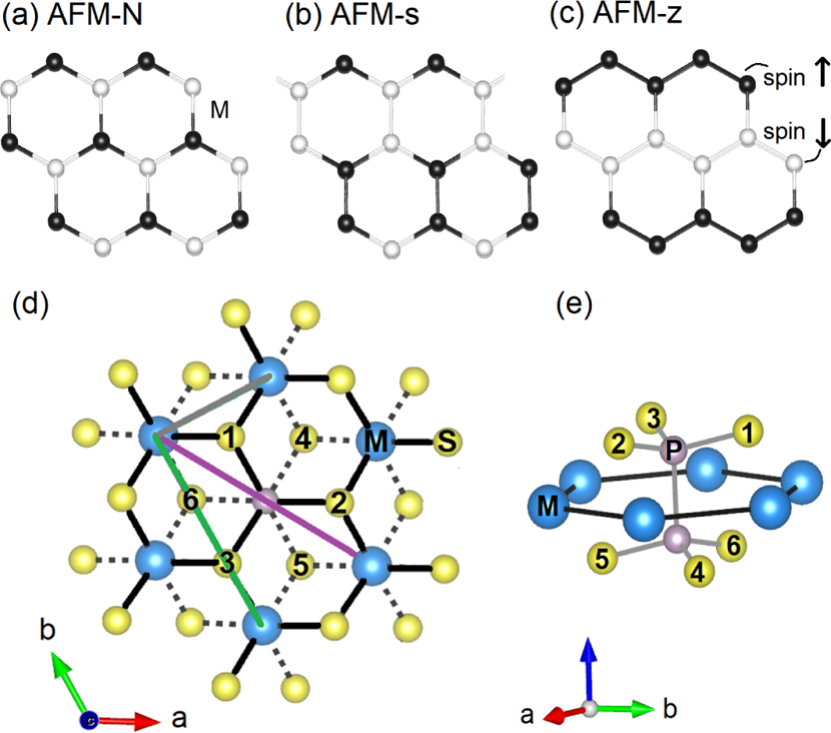
Spin arrangements of the metal atoms in (a) AFM Néel (AFM-N), (b) AFM stripy (AFM-s), and (c) AFM zigzag (AFM-z) states. (d) Top and (e) side views of the crystal structure of the MPX_3_ system with the metal atoms denoted in blue and surrounded by the sulfur atoms (yellow balls). The gray, green, and violet lines indicate the NN, 2NN, and 3NN distance between the metal atoms, respectively. The dotted and solid lines indicate that the sulfur atoms are below and above the metal layer, respectively.

The magnetic ground states of MnPS_3_ and NiPS_3_ exhibit AFM Néel (AFM-N)- and AFM zigzag (AFM-z)-type ordering (see Figure 1), respectively. The neutron diffraction data predicted that the Mn^2+^ (3d^5^, *S* = 5/2) spins are slightly tilted (around 8°) from the perpendicular direction of the honeycomb lattice,^68^ whereas the Ni^2+^ (3d^8^, *S* = 1) spins are aligned within the honeycomb plane. In addition, due to the different filling of the 3d orbitals for various metals ions (Fe, Mn, Ni *etc.*), the size of the magnetic exchange coupling (*J*) also changes. The existence of magnetic ordering at finite temperature in 2D limit requires the magnetic anisotropy in accordance to the Mermin–Wagner theorem. Currently, only FePS_3_, which possesses a strong out-of-plane easy axis, has been experimentally reported to exhibit AFM order in the monolayer up to 118 K.^8^ In order to explain the anisotropic order, the dipolar interactions between the magnetic moments or a single-ion anisotropy resulting from a nonzero spin–orbit coupling should be considered. The recent experimental reports, such as magnon band measurements,^69^ support the claim that the dipolar interactions should be the leading term, whereas the electron spin resonance^70^ and critical behavior measurements^71^ indicate that the single-ion anisotropy might come into play. In addition, experimental observations demonstrated that the AFM ordering persists down to bilayer samples and is suppressed in the monolayer.^72^ Notably, the recent theoretical report^73^ has questioned the Raman criterion used for the monolayer studies therein, suggesting that NiPS_3_ magnetic ordering could be presented in monolayer samples, as also indicated by strong two-magnon continuum existing in thin samples of NiPS_3_.^72^ In the case of MnPS_3_, the magnetic order has been presented down to the bilayer and was reported to be absent in the monolayer.^74^ The suppression of the Néel temperature in thin samples can be associated with reducing the interplanar coupling in atomically thin samples.^3,72^ Aforementioned results demonstrate that the magnetic ordering in monolayers of MPX_3_ is still under a hot debate and many experiments are being carried out to verify the theoretical predictions. Similar effects of strong spin fluctuation and absence of interlayer exchange coupling that weaken the long-range spin order in the 2D limit have been reported in other layered magnets such as Cr_2_Ge_2_Te_6_ and CrI_3_.^3^

Here, we focus on the rationally invariant Heisenberg contribution, whereas the dipolar and single-ion contributions are out of the scope of the present work. The latter has been estimated to 0.3 and 0.009 meV for NiPS_3_ and MnPS_3_, respectively, and discussed theoretically in ref (66). Here, we present the results of exchange couplings up to the third nearest neighbor (for clarification, see Figure S1 in Supporting Information).

The exchange interaction up to the third nearest neighbor (*J*_*i*_^M^) between the metal atoms of the hosts has been widely studied in a series of previous studies^21,66,67,75−77^ (see Table 1). Note that the prediction of the magnetic ground state within the DFT calculations does not depend on Hubbard interaction, whereas it is well known that the exchange coupling strength is sensitive to both *U* and the lattice parameters. We set the Hubbard *U* parameter to *U* = 5 eV and *U* = 6 eV for the 3d orbitals of Mn and Ni atoms, respectively. These values are calculated from first principles using the Cococcioni approach.^55^ Our predicted *J*_*i*_^M^ values are in good agreement with neutron diffraction experiments (see Table 1). The dominant exchange coupling is *J*_3_^Ni^, which is much stronger than *J*_1_^Ni^. Note, that the experimental ratio of critical temperatures  is reflected in the relation of dominant contributions *J*_3_^Ni^ > *J*_1_^Mn^. In both cases, *J*_2_ is much smaller than the other two exchange couplings. In particular, *J*_2_ and *J*_3_ couplings might be considered as superexchange interactions involving the atoms in the path M–S1···S4–M for *J*_2_ (see Figure 1d,e), where the S atoms are located in different sublayers, whereas the *J*_3_ interaction is mediated by S atoms located in the same sublayer through the bridge M–S1···S2–M. One could expect stronger hybridization of the S p states and M 3d states within the same sublayer of S atoms. In addition, the calculations reveal that *J*_1_ is AFM and FM for MnPS_3_ and NiPS_3_, respectively. Note that for both MnPS_3_ and NiPS_3_, the angle between the M–S–M atoms is close to 90° (83.4° for MnPS_3_ and 85.4° for NiPS_3_) for pointing to FM superexchange according to Goodenough–Kanamori–Anderson rules.^78,79^ The direct M–M and indirect M–S–M (superexchange) mechanisms are of crucial importance to understand the differences between these two systems.

**Table 1:** Exchange Coupling Strengths *J*_*i*_^M^ of MnPS_3_ and NiPS_3_ Systems Using Model Calculations and Various Lattice Parameters and On-Site Coulomb Repulsion *U* in the DFT + *U* Calculations^a^

structure	*J*_1_^M^	*J*_2_^M^	*J*_3_^M^	|*J*_2_^M^/*J*_1_^M^|	|*J*_3_^M^/*J*_1_^M^|
MnPS_3_ (model calculations, see caption above)	19.5	0.35	7.76	0.02	0.40
MnPS_3_ (PBE, *a* = 6.00 Å, AFM-N)^66^	1.21	0.18	0.54	0.15	0.45
MnPS_3_ (*U* = 5 eV, *a* = 6.11 Å, AFM-N) this work	1.04	0.05	0.53	0.05	0.51
MnPS_3_ (*U* = 5 eV, *a* = 5.88 Å, AFM-N)^67^	1.58	0.08	0.46	0.05	0.29
MnPS_3_ (*U* = 4 eV, *a* = 6.00 Å, AFM-N)^21^	0.4	0.03	0.15	0.08	0.38
MnPS_3_ (*U* = 3 eV, *a* = 6.00 Å, AFM-N)^66^	1.42	0.08	0.52	0.06	0.37
MnPS_3_ (experiment, AFM-N)^64^	1.54	0.14	0.36	0.09	0.23
NiPS_3_ (model calculations, see caption above)	–4.9	0.06	14.8	0.01	3.0
NiPS_3_ (PBE, *a* = 5.78 Å, AFM-z)^21^	–11.3	–0.12	36	0.01	3.2
NiPS_3_ (*U* = 6 eV, *a* = 5.84 Å, AFM-z) this work	–3.34	–0.19	13.7	0.06	4.1
NiPS_3_ (*U* = 4 eV, *a* = 5.78 Å, AFM-z)^21^	–4.11	1.95	17.4	0.47	4.2
NiPS_3_ (*U* = 3 eV, *a* = 5.78 Å, AFM-z)^66^	–2.6	–0.32	14	0.12	5.4
NiPS_3_ (experiment, AFM-z)^65^	–3.8	0.2	13.8	0.05	3.6

a Positive (negative) *J*_*i*_^M^ indicate AFM (FM) correlations, respectively. Note, that in refs (64) and (65), different conventions of the exchange couplings *J*_*i*_^M^ were used. The AFM-N and AFM-z indicate the magnetic ground state of the system. The exchange couplings in the model calculations are obtained within the direct exchange mechanism [for MnPS_3_ using eqs 2 and 4 whereas for NiPS_3_ using eq 3]—except for *J*_1_^Ni^ for NiPS_3_, which contains also an important superexchange contribution and follows from eq 7; see text for further details. More details on the calculation of the magnetic exchanges within the DFT + *U* approach are present in Supporting Information. All values are given in meV.

### Effective Direct Exchange and AFM Couplings

In order to understand the origin of the exchange couplings and their relative strengths, we consider model Hamiltonians for the direct and superexchange interactions. Note, that the direct exchange discussed in this work is a (second order) kinetic exchange process, which involves only the hopping between the transition metal ions, with the ligands not explicitly involved, *cf.* ref (80), whereas a superexchange term is a fourth- (or higher-) order kinetic exchange process, which explicitly involves the hopping over the ligands, *cf.* ref (80). In addition, we are pointing out below the mechanisms which could impact the sign of *J*_1_.

To understand the origin of the magnetic couplings, we first write down a minimal many-body model which solely contains the valence electrons of the transition metal ion^81^—the multi-band Hubbard model. From this, using the second-order perturbation theory that is valid in the Mott insulating limit, we derive the (effective) direct exchange processes. By construction, all obtained spin couplings have to be AFM. Therefore, while surprisingly successful, the following simple analysis will not be able to explain the onset of the FM couplings in NiPS_3_ (more on this at the end of the section).

**Figure 2:**
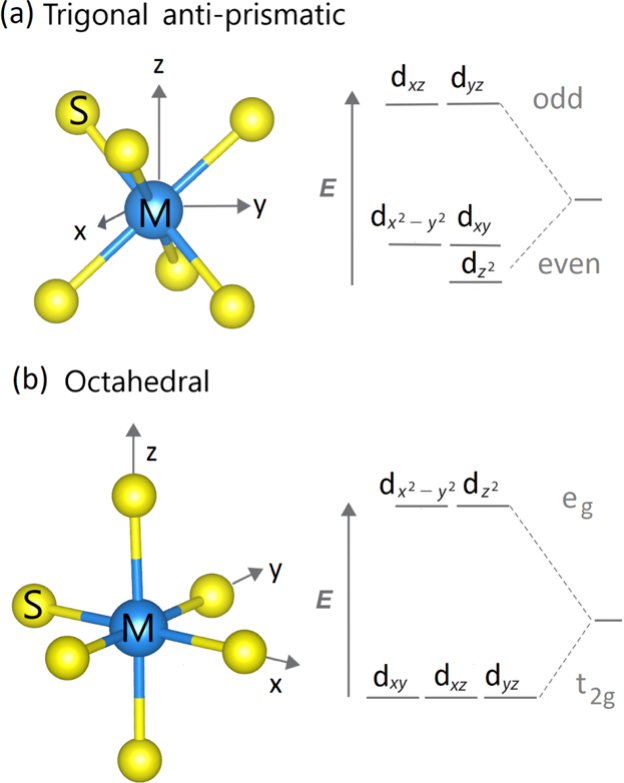
(a) Trigonal anti-prismatic and (b) octahedral geometries. On the right side, the d-orbital splitting of the metal atom in the corresponding crystal fields is shown. M and S denote the metal atom and sulfur atom, respectively.

In the monolayers of MnPS_3_ and NiPS_3_, the metal atoms are surrounded by six sulfur atoms (MS_6_ octahedron) and exhibit *D*_3*d*_ point group symmetry in trigonal anti-prismatic environment of the ligands (sulfur atoms), see Figure 2a. Hence, due to this trigonal crystal field effect, the d manifold splits in two disentangled subsets of bands. The only coupling between these subsets is the spin–orbit coupling.^82^ The bands lower in energy are even (d_*x*^2^–*y*^2^_, d_*xy*_, and d_*z*^2^_) with respect to the basal plane, while the bands higher in energy are odd (d_*xz*_ and d_*yz*_) with respect to the basal plane. The Mn ion is d^5^ and Mn d bands split into half-filled even and half-filled odd bands; therefore, the magnetic coupling acquires contributions both from the even and the odd orbitals. Instead, because the Ni ion has a d^8^ configuration, the even orbitals are fully occupied, and therefore, there is no magnetic contribution from these orbitals. Altogether, the minimal model is the five-band Hubbard model with a simplified structure of the Coulomb interactions (no spin on-site spin exchange and pair-hopping terms, *cf.* ref (83))
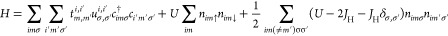
1In this model, *c*_*im*σ_^†^ creates an electron with spin σ = ↑, ↓ in a Wannier orbital |*m*⟩ = |*x*^2^ – *y*^2^⟩, |*xy*⟩, |*xz*⟩, |*yz*⟩, or |3*z*^2^ – *r*^2^⟩ at site *i*, and *n*_*im*σ_ = *c*_*im*σ_^†^*c*_*im*σ_. ↑ (↓) indicates the spin up (down). The parameter *t*_*m*,*m*′_^*i*,*i*′^ is the hopping integral from orbital *m* at site *i* to orbital *m*′ at site *i*′. The on-site terms *t*_*m*,*m*′_ = ε_*m*,*m*′_ give the crystal field splitting. *U* and *J*_H_ are the direct and (Hund) exchange terms of the screened on-site Coulomb interaction.

We applied second-order perturbation theory in *t*/*U* and for the commensurate electron filling of Mn and Ni ions. The direct exchange constant for the valence electrons occupying the Mn odd orbitals is
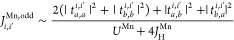
2where *a* and *b* are the dxz and d_*yz*_ orbitals, respectively, while *i* and *i*′ are the Mn lattice sites. In this formula, we take in consideration both the cases with *i* = 1 ≠ *i*′ = 2 and *i* = *i*′ = 1. *U*^Mn^ and *J*_H_^Mn^ are the Coulomb repulsion and Hund coupling, respectively, in the case of the Mn atoms. The Hund’s rule interaction between odd and even electrons yields a magnetic coupling between these electrons; therefore, the denominator depends on the occupancy of the even orbitals. By symmetry, the on-site energies are ϵ_*a*_^Mn1^ = ϵ_*a*_^Mn2^ = ϵ_*b*_^Mn1^ = ϵ_*b*_^Mn2^.

Similarly, we obtain the direct exchange constant for the valence electrons occupying the odd orbitals of the Ni atoms
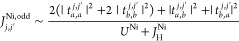
3where *a* and *b* are the d_*xz*_ and d_*yz*_ orbitals, respectively, while *j*, *j*′ are the Ni lattice sites. In this formula, we take in consideration both the cases with *j* = 1 ≠ *j*′ = 2 and *j* = *j*′ = 1. *U*^Ni^ and *J*_H_^Ni^ are the Coulomb repulsion and Hund coupling, respectively, in the case of the Ni atoms. By symmetry the on-site energies are ϵ_*a*_^Ni1^ = ϵ_*a*_^Ni2^ = ϵ_*b*_^Ni1^ = ϵ_*b*_^Ni2^.

Finally, the direct exchange constant for the even Mn orbitals is as follows
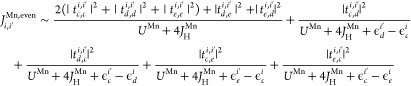
4where *c*, *d* and *e* are the orbitals *d*_*z*^2^_, d_*x*^2^–*y*^2^_, d_*x,y*_ respectively, while *i* and *i*′ are the metal lattice sites. In this formula, we take in consideration both the cases with *i* = 1 ≠ *i*′ = 2 and *i* = *i*′ = 1. *U*^Mn^ and *J*_H_^Mn^ are the Coulomb repulsion and Hund coupling, respectively, in the case of the Mn atoms. By symmetry the on-site energies are ϵ_*c*_^Mn1^ = ϵ_*c*_^Mn2^ and ϵ_*d*_^Mn1^ = ϵ_*d*_^Mn2^ = ϵ_*e*_^Mn1^ = ϵ_*e*_^Mn2^.

The obtained (total) direct exchange, *J* = *J*^odd^ + *J*^even^, is positive for both Mn and Ni ion and for any distance *j* – *j*′. Hence, as already mentioned, it is always AFM by construction.

### Numerical Evaluation of the AFM Couplings

The Wannier basis provides us with *ab initio* values of the hopping integrals and crystal field splitting. We calculate the hopping parameters and the on-site energies using the interpolated band structure of the Wannier functions of the d-subsector. The on-site energies for Mn and Ni are: ϵ_*a*_^Mn1^ = −1197.7 meV, ϵ_*c*_^Mn1^ = −2082.6 meV, ϵ_*d*_^Mn1^ = −2179.3 meV, and ϵ_*a*_^Ni1^ = −1768.5 meV. We have three first nearest neighbors (1NN), six second nearest neighbors (2NN), and three third nearest neighbors (3NN). In the case of odd orbitals, the 3NN couplings are greater than the 1NN couplings, even by an order of magnitude; therefore, it is very important to consider these hopping amplitudes in the calculations. On the other hand, for even orbitals, the 1NN couplings are greater than the 3NN couplings. The 2NN couplings are always smaller with respect to the 1NN couplings and 3NN couplings. In the case of even orbitals, we neglect the difference between the on-site energies assuming the following approximation ϵ_*c*_^Mn^ – ϵ_*d*_^Mn^, ϵ_*c*_^Mn^ – ϵ_*e*_^Mn^ ≪ (*U*^Mn^ + 4*J*_H_^Mn^).

Now, we will numerically evaluate the second- and third-neighbor direct exchange as a function of the first-neighbor direct exchange. For the odd subsector of MnPS_3_, we obtain *J*_2_^Mn,odd^ = 0.037*J*_1_^Mn,odd^ and *J*_3_^Mn,odd^ = 2.026*J*_1_^Mn,odd^. For the even subsector of the MnPS_3_, we obtain *J*_2_^Mn,even^ = 0.015*J*_1_^Mn,even^ and *J*_3_^Mn,even^ = 0.016*J*_1_^Mn,even^. Considering that *J*^Ni,even^ = 0 due to fully occupied even orbitals, for the odd subsector of NiPS_3_, we obtain *J*_2_^Ni,odd^ = 0.047*J*_1_^Ni,odd^ and *J*_3_^Ni,odd^ = 11.09*J*_1_^Ni,odd^. If we consider the total direct exchange value as the sum of the odd and even sector, we have *J*_1_^Mn^ = (152.4 eV·meV)/(*U*^Mn^ + 4*J*_H_^Mn^) for the first-neighbor coupling in MnPS_3_. For the second and third neighbors, we obtain *J*_2_^Mn^ = 0.018*J*_1_^Mn^ and *J*_3_^Mn^ = 0.397*J*_1_^Mn^, and therefore, for MnPS_3_, the dominant direct exchange comes from the first-neighbor coupling. Using a Coulomb repulsion of 5 eV for Mn and a Hund coupling of 0.7 eV, we obtain the numerical values equal to *J*_1_^Mn^ = 19.5 meV, *J*_2_^Mn^ = 0.35 meV, and *J*_3_^Mn^ = 7.76 meV, (see Table 1). Note that the values are overestimated in comparison to experimental values (see Table 1); however, the signs and the dominant contributions are the same.

When we numerically evaluate the direct exchange of NiPS_3_, we obtain *J*_1_^Ni^ = (8.957 eV·meV)/(*U*^Ni^ + *J*_H_^Ni^) for the first-neighbor coupling, *J*_2_^Ni^ = 0.047*J*_1_^Ni^ and *J*_3_^Ni^ = 11.09*J*_1_^Ni^ for the second and third neighbors, respectively. Remarkably, the 3NN magnetic direct exchange is larger than the 1NN exchange in the odd case. Using a Coulomb repulsion of 6 eV for Ni and a Hund coupling of 0.7 eV, we obtain the numerical values equal to *J*_1_^Ni^ = 1.3 meV, *J*_2_^Ni^ = 0.06 meV, and *J*_3_^Ni^ = 14.8 meV, where last two are reported in Table 1. Note, that *J*_2_^Ni^ and *J*_3_^Ni^ are in good agreement with experimental values (see Table 1), while *J*_1_^Ni^ is significantly different and has a wrong sign. Therefore, in the case of *J*_1_^Ni^, a more complex superexchange model will be presented further in the text.

Altogether, we obtain that the simple direct exchange scheme gives *J*_3_^Ni^ ≫ *J*_1_^Ni^ ≫ *J*_2_^Ni^ and *J*_1_^Mn^ > *J*_3_^Mn^ ≫ *J*_2_^Mn^. In the Ni case, the leading term is *J*_3_^Ni^, while in the Mn case, the leading term is *J*_1_^Mn^. The reason for this different behavior comes from the different filling that produces *J*^Ni,even^ = 0. The calculated direct exchange couplings are qualitatively in agreement with the magnetic couplings obtained experimentally or using DFT—except that *J*_1_^Ni^ and *J*_2_^Ni^ are FM due to more complex magnetic exchange not taken into account by the (simple) direct exchange scheme (see below). Even though the latter coupling is relatively small, note that considering in more detail, such discrepancy is relevant for an accurate description of the magnetic coupling and, hence, of the magnetic critical temperature. The different leading exchange terms in Mn and Ni compounds open the way to manipulate the magnetism by tuning the concentration of Mn and Ni compounds or by adding new magnetic materials as dopants.

### Superexchange and FM Coupling in NiPS_3_

So far, we considered an effective direct exchange model, which solely contained the exchange processes due to the electrons hopping between the transition metal (Mn or Ni) ions. Note that such a model should be considered an effective one, for in reality, the hopping between the neighboring Mn or Ni ions is predominantly mediated by the sulfur ions. Hence, a natural extension of the direct exchange model should explicitly contain the exchange processes on the sulfur ions—in fact, it is such a nearest neighbor superexchange model^84^ that is studied below to explain the onset of the nearest neighbor FM exchange in NiPS_3_. Note that for consistency, we comment at the end of this subsubsection why the more complex superexchange model is not needed to understand the other, the first- and third-neighbor,^a^ magnetic couplings in MPS_3_—that is, the effective direct exchange model is enough.

**Figure 3:**
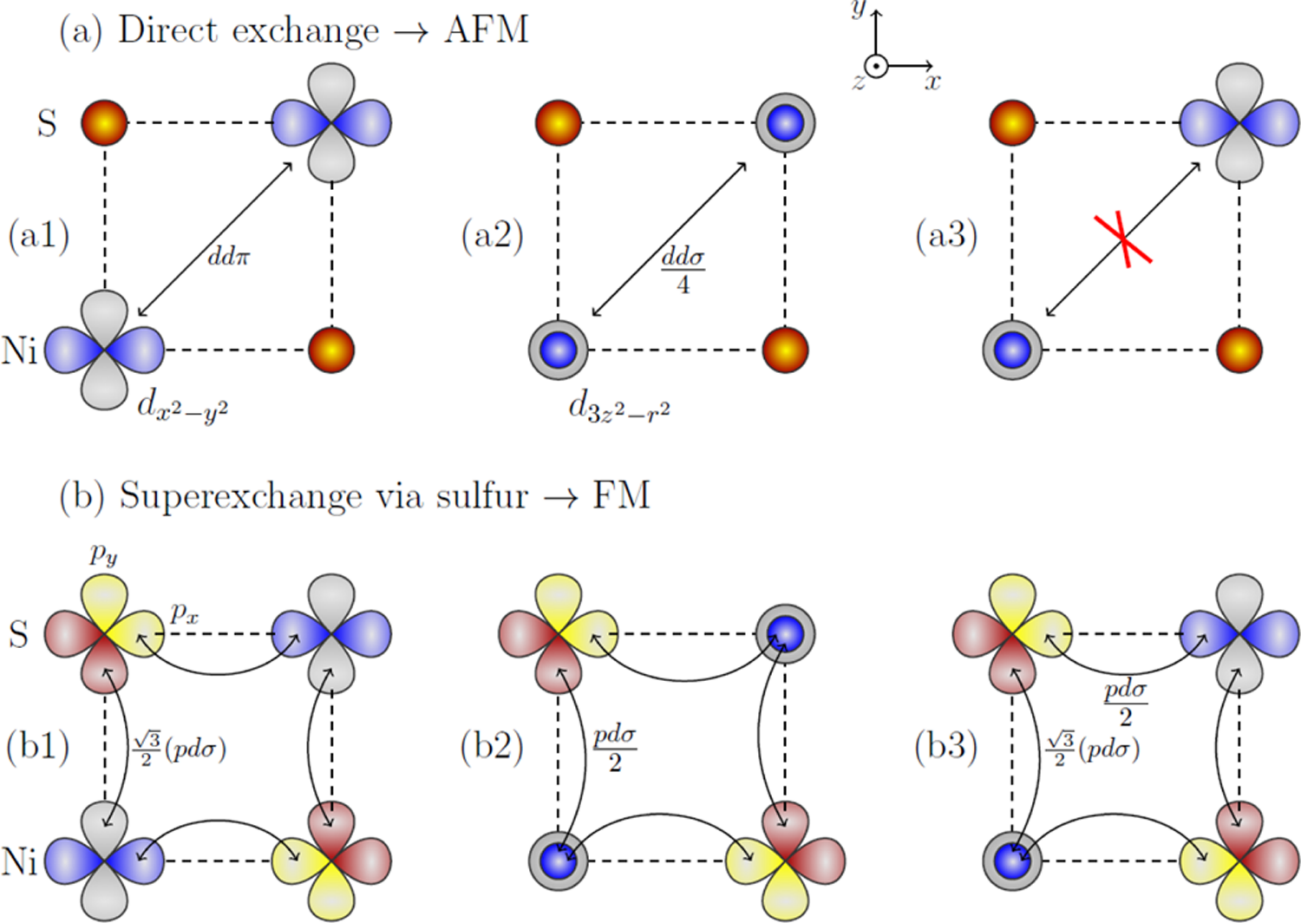
Schematic comparison between the AFM direct exchange (top panels) and the FM superexchange (bottom panels) processes in NiPS_3_. Finite direct-AFM exchange processes due to (a1) nonzero hopping elements ∝ *dd*π between the nearest-neighbor d_*x*^2^–*y*^2^_ orbitals on nickel and (a2) nonzero hopping elements ∝ *dd*σ between the nearest-neighbor d_3*z*^2^–*r*^2^_ orbitals on nickel. Lack of direct-AFM exchange processes due to vanishing hopping elements between the nearest-neighbor d_*x*^2^–*y*^2^_ and d_3*z*^2^–*r*^2^_ on nickel. Finite FM superexchange processes due to (b1) nonzero hopping elements ∝ *pd*σ between the nearest-neighbor d_*x*^2^–*y*^2^_ orbitals on nickel and the nearest-neighbor p_*x*_/p_*y*_ orbital on sulfur, (b2) nonzero hopping elements ∝ *pd*σ between the d_3*z*^2^–*r*^2^_ orbitals on nickel and the nearest-neighbor p_*x*_/p_*y*_ orbital on sulfur, and (b3) nonzero hopping elements ∝ *pd*σ between the d_*x*^2^–*y*^2^_ orbital on nickel and the nearest-neighbor p_*x*_/p_*y*_ orbital on sulfur and the d_3*z*^2^–*r*^2^_ orbital on nickel and the nearest-neighbor p_*x*_/p_*y*_ orbital on sulfur. See text for more details.

We introduce the nearest neighbor superexchange model^84^ for NiPS_3_ by considering two Ni ions connected *via* two sulfur atoms over two 90° bonds, see Figure 3. Note that, because Ni^2+^ is in a d^8^ configuration and sulfur ions are fully occupied, it is easier to consider the hole language as discussed below. Moreover, in what follows, we neglect the small trigonal distortions and we assume an octahedral crystal field with the division in t_2g_ and e_g_ orbitals [with the coordinate system defined in such a way that the *xy* plane coincides with the plane formed by the nearest-neighbor transition metal ion and sulfur, see Figures 2b and 3]. Hence, we begin by considering a fully atomic limit without hopping (zeroth order of perturbation theory in the small kinetic energy) in which there are two holes localized in two distinct Ni e_g_ orbitals (d_*x*^2^–*y*^2^_, d_3*z*^2^–*r*^2^_; in what follows assumed to be energetically degenerate, see also above) and two (lying higher by the charge transfer energy Δ) empty p orbitals (p_*x*_, py) on sulfur. As before, due to the strong Hund’s rule *J*_H_, the two Ni^2+^ holes form a high spin *S* = 1 state. Now, let us perform a perturbation theory in the kinetic energy (over Coulomb repulsion *U* and charge transfer energy Δ) and consider the possible exchange processes—which are of two kinds:

First, there are direct exchange processes between the nickel ions, see Figure 3a. By definition, these concern virtual occupancies of one of the nickel ions by three holes [with a relative energy cost of *U* + *J*_H_ according to the simplified structure of the Coulomb interactions, see eq 1] and are possible once the hole can directly hop back and forth between the nickel orbitals under consideration. According to the Slater–Koster scheme,^85^ which is qualitatively confirmed by our DFT calculations, the latter is allowed between the pair of d_*x*^2^–*y*^2^_ orbital (*dd*π hopping element) and between the pair of *d*_3*z*^2^–*r*^2^_ orbitals (*dd*σ/4 hopping element; the small *dd*δ element can be neglected), *cf.*Figure 3(a1,a2); note that the hopping between the d_*x*^2^–*y*^2^_ and the d_3*z*^2^–*r*^2^_ orbital vanishes in this geometry, *cf.*Figure 3(a3). Altogether we obtain the nearest-neighbor direct exchange contribution
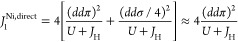
5where we assumed a typical relation between the Slater–Koster hopping integrals *dd*σ ≈ 2*dd*π. It is important to state here that the above direct exchange process is different than the *effective* direct exchanges process defined in the previous subsections—for the latter ones may include all indirect hoppings (*e.g., via* sulfur) between the two nearest neighbor nickel ions.

Second, there are superexchange processes between the nickel ions, see Figure 3b. By definition these concern virtual occupancies of one of the sulfur ions by two holes (with an energy cost associated with the charge transfer 2Δ for antiparallel spins or 2Δ – *J*_H_ for parallel spins^b^) and are possible once the two holes hop back and forth between the sulfur and nickel orbitals under consideration. Again, using the Slater–Koster scheme,^85^ and considering the three distinct possible hopping processes shown in Figure 3(b1–b3), we obtain the nearest neighbor superexchange contribution
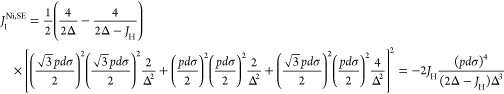
6which is negative (FM) due to the lowering of the energy for a FM (virtual) occupancy of sulfur by two holes in the superexchange process. Note that for a given Ni–Ni bond, there are always two superexchange processes: one over the top-left and one over the bottom-right sulfur (hence the factor of two within the square brackets in the above formula). Moreover, the process depicted in Figure 3(b3) has to be multiplied by a factor of two, for one can interchange the position of the d_*x*^2^–*y*^2^_ and the d_3*z*^2^–*r*^2^_ orbital and in this way double the amplitude of this process. Finally note that, due to the fact that we have spins *S* = 1 on nickel, overall the above superexchange process is reduced by a factor 1/2 with respect to to an analogous one for the *S* = 1/2 on copper (the superexchange processes have to be projected on the high-spin *S* = 1 states on both nickel ions—hence a factor of  reduction).

Let us now comment why the above FM superexchange mechanism is not important for the nearest neighbor exchange in MnPS_3_. The reason for this is that in the case of manganese ions, we are a bit closer to the situation discussed in ref (84), which, for instance, shows the AFM exchange coupling in the case of half-filled t_2g_ subshells of Cr^3+^ in LiCrS_2_. More precisely, the situation for the Mn^2+^ ions is as follows. On one hand, the AFM direct exchange is much stronger for Mn^2+^ because one of the t_2g_ electrons (the d_*xy*_) can hop over the *dd*σ bond. On the other hand, the superexchange also contains an additional strong^84^ AFM contribution due to the superexchange processes over one p_*z*_ sulfur orbital—which strongly hybridizes with the two nearest t_2g_ orbitals. Altogether, as confirmed by the effective direct exchange studies in the previous subsection, these two mechanisms originating in the t_2g_ exchange processes easily overcome the (above-described) FM processes for the e_g_ orbitals.

Finally, we mention that the (surprisingly) strong third-neighbor coupling in MPS_3_ can easily be explained using the superexchange model. First, for the t_2g_ sector, the strong AFM third-neighbor coupling is already discussed in ref (84)—see Figure 7 of the reference. Second, one can easily imagine that a similarly strong AFM coupling can also be realized for the e_g_ electrons: in order to understand it, one just needs to replace the two third-neighbor d_*xy*_ orbitals with the d_*x*^2^–*y*^2^_ orbitals and rotate the sulfur p orbitals by 90° in the process shown in Figure 7 of ref (84). In fact, the latter one should have a very high amplitude and hence a really strong third-neighbor exchange in NiPS_3_.

### Numerical Evaluation of the FM Coupling in NiPS_3_

Having derived the direct exchange and superexchange processes between the nearest neighbor nickel ions, we are now ready to estimate the contributions of these both (competing) spin interaction terms. To this end, we assume that Coulomb repulsion *U* = 6 eV (the chosen value of our DFT + *U* approach, see Table 1) and take a typical (for 3d transition metal compounds) value of Hund’s exchange *J*_H_ = 0.7 eV. Next, based on the DFT calculations, we estimate that (i) the charge transfer energy Δ = 3 eV, (ii) the hopping *pd*σ = 0.9 eV, and (iii) the hopping *dd*σ = 0.05 eV. From this and using eqs 5 and 6, we can easily calculate the AFM and FM contributions to the spin exchange
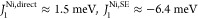
7As the second exchange is larger (by absolute value), we conclude that it is a relatively strong superexchange along the 90° nickel-sulfur-nickel bonds, which triggers the FM exchange along the nearest neighbor nickel ions. The sum of the aforementioned contributions leads to FM exchange coupling (*J*_1_^Ni^ = −4.9), which is in good agreement with experimental studies (see Table 1).

At this point, we would like to comment on the fact that for the edge-sharing copper chains, typically one entirely neglects the direct exchange *cf.* ref (86). The reason for this is that, in the copper chains, the direct copper hopping *dd*σ = 0.08 eV,^87^ whereas usually one assumes that for the cuprates, *pd*σ ≈ 1.5 eV. With such hopping parameters (and assuming the typical cuprate values of *U* = 8 eV, Δ = 3 eV, and oxygen *J*_H_ = 0.7 eV), one can immediately see that for the copper chains with 90° geometry, *J*^Cu,direct^ ≈ 3 meV ≪ |*J*^Cu,SE^| ≈ 50 meV—which justifies why the direct copper exchange is typically neglected in such studies. In reality, the FM exchange is actually really small in such copper chains due to the angle along the copper–oxygen–copper bond not being strictly equal to 90°.

A strong suppression of the direct exchange is also observed in CrI_3_,^88^ which is an experimentally confirmed 2D ferromagnet. CrI_3_ is isostructurally identical to MPS_3_ compounds when P atoms are removed and has partially-filled t_2g_ shells, just as MnPS_3_. This might suggest that CrI_3_ should predominantly have AFM interactions, just as MnPS_3_. However, this is not the case because the relative strength of the direct and superexchange mechanisms is different in CrI_3_ and MnPS_3_. In CrI_3_, the Cr–I–Cr bond angle is very close to 90°, leading to a strong FM superexchange, while the Cr–Cr distance is relatively large (3.95 Å), giving rise to a very weak AFM direct exchange. Interestingly, due to the larger distances between the magnetic atoms in CrI_3_, the third-neighbor exchange is also less relevant.

### Doped Systems (M_3/4_,X_1/4_)PS_3_

**Figure 4:**
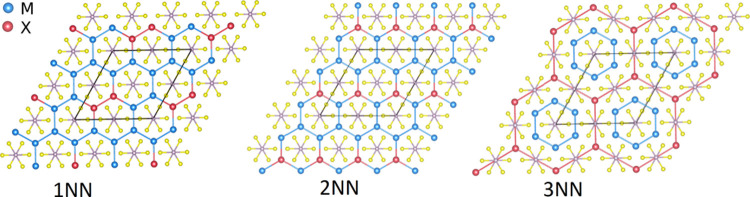
Crystal structure of 2 × 2 supercells consisting of fourfold primitive unit cells. The M and X are the transition metals of the host and dopant atoms, respectively. 1NN, 2NN, and 3NN denote the nearest neighbors, second neighbors, and third neighbors of the dopants, respectively.

Our strategy is to try to induce long-range FM order *via* chemical doping, keeping the two-dimensional structure of the mother compounds intact. In principle, the chemical doping could influence the magnetic exchanges, especially the long-range ones. To this end, we consider doping of the host systems with distinct possible 3d elements. In particular, at low doping concentration, the main exchange couplings are the magnetic exchanges between the host magnetic atoms (*J*_*i*_^M^ for *i* = 1, 2, 3) and between the host impurity atoms (*J*_*i*_^MX^ for *i* = 1, 2, 3). Although for most of the 3d doping, the Mn–Mn and the Ni–Ni magnetic couplings remain AFM, in the case of Cr doping, the AFM exchanges between the magnetic atoms of the host turn into FM ones.^89^ Thus, we performed DFT + *U* calculations for 25% concentration of the magnetic dopants (X = Cr, Mn, and Ni) in the hosts of NiPS_3_ and MnPS_3_. We have employed various structural arrangements of the atoms (see Figure 4) and collinear spin configurations such as AFM Neel (AFM-N), zigzag (AFM-z), stripy (AFM-s), and FM case. For each of the configurations, the atomic positions have been fully optimized, keeping the lattice constants equal to the pure optimized monolayer structures (see Methods).

**Figure 5:**
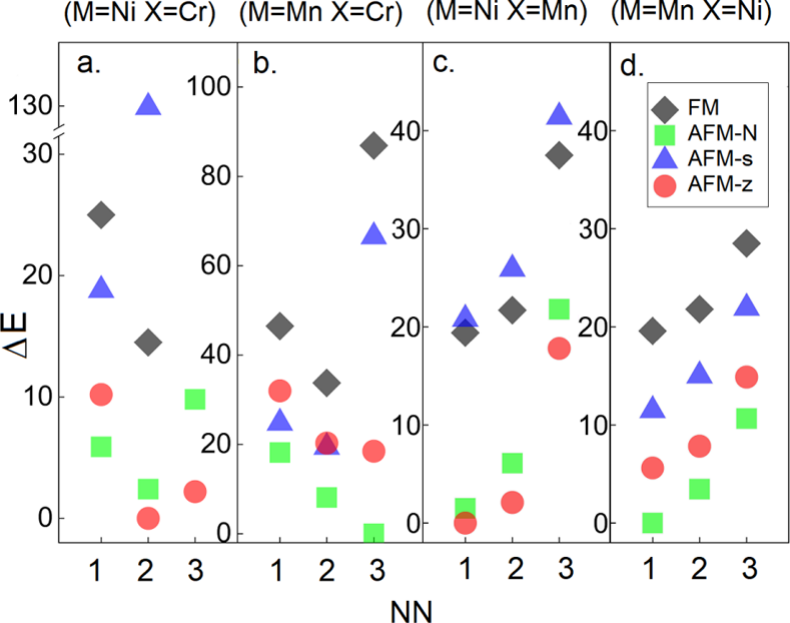
(a–d) Energy difference (Δ*E*) between a particular magnetic configuration (gray rhombus FM, green square AFM-N, blue triangle AFM-s, and red circle AFM-z) and the magnetic ground state for each of the alloys (M_3/4_,X_1/4_)PS_3_. Note, that in each of the plots, the most energetically preferable arrangement of the dopants is at the lowest energy. The energy is given in eV per magnetic atom for various structural arrangements of the dopant in the host (1NN, 2NN, and 3NN).

**Figure 6:**
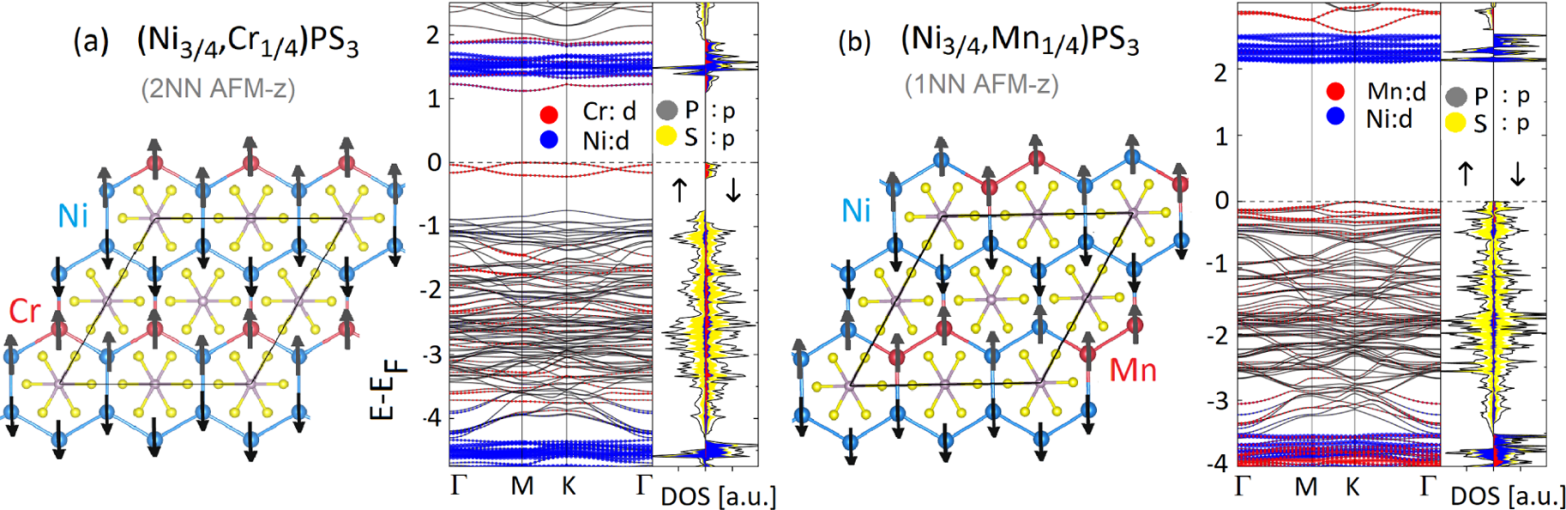
Band structure and projected density of states (PDOS) for 2D ferrimagnetic alloys: (a) (Ni_3/4_,Cr_1/4_)PS_3_ and (b) (Ni_3/4_,Mn_1/4_)PS_3_. On the left side of (a,b), the corresponding atomic structural arrangements and local spin magnetic configurations are presented. Note that the spin of the dopant and the host atoms has different magnitudes, which are not visible here (spin arrows are not in scale). For better visibility, the spin channels and projections of the p states of S and P atoms are presented only in PDOS. The energy is given in eV, and the Fermi level is placed at zero.

Let us first discuss the impact of the dopant on the energetic and structural properties of the hosts. The energy difference between a particular configuration and the magnetic ground state for each considered alloy is presented in Figure 5. Note that each of the employed alloys preserves the magnetic ground state of the host, independent of the structural arrangement of the impurity and the type of the dopants (see Figure 5). In addition, the favorite Mn and Ni dopant position is at 1NN (see Figure 5c,d), whereas the Cr dopants prefer to lie further apart, in particular, at 2NN (see Figure 5a) and 3NN (see Figure 5b) structural positions for NiPS_3_ and MnPS_3_ hosts, respectively. This reveals that the Mn and Ni dopants have tendency to cluster, while the Cr ions prefer to spread over the host. From now on, we only discuss the magnetic ground-state configurations of the alloys (the one which exhibits the lowest energy in Figure 6 for particular alloy). The magnetization and the band gaps of these systems are collected in Table 2. All considered alloys exhibit a semiconducting behavior (see Table 2). However, only (Ni_3/4_,Cr_1/4_)PS_3_ and (Ni_3/4_,Mn_1/4_)PS_3_ alloys have a nonzero net magnetization. Thus, we focus our discussion on these two ferrimagnetic systems. Owing to the different spins of the host and dopant atoms and particular arrangement of the dopants in the NiPS_3_ host, the ferrimagnetic state appears in these systems. The band structure and the orbital projections of these two ferrimagnetic alloys are presented in Figure 6. Note that in the (Ni_3/4_,Cr_1/4_)PS_3_ case, bands close to the Fermi level are mainly composed of 3d states of Cr dopants, causing a sizeable reduction in the energy gap compared to pure NiPS_3_ (2.3 eV for *U* = 6 eV). In the case of Mn impurities, the valence band maximum is mainly composed of Mn 3d states, whereas the conduction band minimum consists of very flat bands of Ni atoms (see Figure 6b).

**Table 2:** Results for the Most Energetically Favorable Position of the Dopants in the (M_3/4_,X_1/4_) Alloys^a^

alloy (M_3/4_,X_1/4_)PS_3_	magn. state (2 × 2) sc	*M* [μ_B_] per sc	band gap [eV]
(Mn_3/4_,Cr_1/4_)PS_3_	3NN AFM-N	0	1.7 (D, Γ)
(Mn_3/4_,Ni_1/4_)PS_3_	1NN AFM-N	0	2.16 (D at K)
(Ni_3/4_,Cr_1/4_)PS_3_	2NN AFM-z	3.9	1.1 (↑, D) 2.11 (↓, ID)
(Ni_3/4_,Mn_1/4_)PS_3_	1NN AFM-z	5.7	2.11 (for both spins)

a *M* is the total magnetization of the cell.

Next, we examine the mixed exchange couplings between the metal atom of the host and the impurity *J*_*i*_^MX^ within the classical Ising Hamiltonian in the honeycomb lattice for the smallest possible cell containing 25% dopant concentration (for details see Supporting Information and Figure S1 therein). Note that the FM exchange couplings are obtained for the Mn and Ni nearest neighbors and Cr–Cr ions at the second-nearest-neighbor distance (see *J*_2_^X^ in Table 3). The similar results for Cr have been recently reported in ref (90). In addition, the different values for *J*_1_^MnNi^ (−1 meV) and *J*_1_^NiMn^ (−0.7 meV) stems from the different lattice parameters of the hosts (see Table 3). Although the mixed exchange coupling obtained for (Ni_3/4_,Mn_1/4_)PS_3_ correctly reflected the 1NN AFM-z ground-state configuration for this system, the difference between the 1NN AFM-N and 1NN AFM-z is just 1.5 meV per magnetic ion (see Figure 5c), thus resulting in magnetic frustration between the Mn and Ni atoms at the honeycomb sites due to the competition between the Néel and zigzag configurations, similar to the results reported in ref (35). Moreover, the critical temperature is directly related to the strength of the exchange couplings. Generally, the mixed exchange couplings are smaller than those in the corresponding metal atoms of the hosts. Thus, the critical temperature of the mixed structure is expected to be smaller than that for the corresponding pure system, which is in line with recent experimental reports on the Ni_1–*x*_Mn_*x*_PS_3_ alloy^35^ and with series of the mixed-system studies, where the suppression of *T*_N_ temperature with dopant substitution is reported.^27,29,34^ In addition, one should expect the further reduction of *T*_N_ temperature of the employed mixed systems due to the possible disorder of the dopant atoms in the host, as reported in refs (30) and (34), which is not accounted in our studies.

**Table 3:** Exchange Coupling Strengths *J*_*i*_ Calculated for Various Alloy Systems, Implied by the Ising Model^a^

(M_0.75_,X_0.25_)PS_3_	*J*_1_^XM^	*J*_2_^XM^	*J*_2_^X^	*J*_3_^XM^
(Mn_3/4_,Cr_1/4_)PS_3_	2.5	0.1	–1.1	–0.4
(Mn_3/4_,Ni_1/4_)PS_3_	–0.7	–0.5	–0.6	2.0
(Ni_3/4_,Mn_1/4_)PS_3_	–1.0	0.3	0.15	4.9
(Ni_3/4_,Cr_1/4_)PS_3_	2.2	0.4	–0.3	2.3

a Positive and negative *J*_*i*_ indicate AFM and FM ordering, respectively.

## Conclusions

*First*, we examined the magnetic and electronic properties of the AFM-ordered systems MnPS_3_ and NiPS_3_ (without magnetic impurities). We presented a qualitative explanation for the relative ratio of the different nearest neighbor (first, second, and third neighbor) exchange couplings by studying an effective direct exchange for both MnPS_3_ and NiPS_3_. In particular, we demonstrated that the third-neighbor exchange dominates in NiPS_3_ due to the filling of the t_2g_ subshell, whereas for MnPS_3_, the first-neighbor exchange is prevailed, owing to the presence of the t_2g_ magnetism.

We showed in this work that the onset of the nearest-neighbor FM coupling in NiPS_3_ is due to the relatively strong (FM) superexchange, which is enabled by the complete filling of the t_2g_ shell and by the (close to) 90° nickel–sulfur–nickel nearest neighbor bond. Nevertheless, even these relatively “fortunate” circumstances do not guarantee that the strong further neighbor exchange can also be FM. The reason for this lies in the further neighbor bonds, which are no longer of a “90° variety”—and the latter bonds are, apart from specific Jahn–Teller effects, the only way to obtain FM couplings in Mott insulators. From a more general perspective, this study confirms the paradigm that the AFM couplings are natural to the Mott insulating compounds.

*Second*, we examined the properties of the MnPS_3_ and NiPS_3_ compounds doped with 25% impurities (Ni in MnPS_3_, Mn in NiPS_3_, and Cr in both hosts). It turned out that all of the investigated alloys are Mott insulating, albeit with generally smaller band gaps than those of the corresponding host. Crucially, we demonstrated an extreme robustness of the AFM phases against impurity doping of the MnPS_3_ and NiPS_3_ compounds. Hence, ferromagnetism cannot be easily stabilized by impurity doping, in agreement with the above paradigm. Nevertheless, as the dopants have different spins than the pure phases, the alloys exhibit ferrimagnetic properties for particular arrangements of dopants.

The Mn and Ni impurities prefer to form dimers within the host, whereas the Cr dopants prefer to be further apart. Interestingly, unlike for the hosts, the first and second (dopant–host) exchange couplings are of similar order of magnitude. The latter leads to frustration in the case of AFM couplings. We suggest that this may be one of the reasons of the observed lower magnetic ordering temperature of the doped systems.^35^

Our work sheds light on the origin of magnetism in the AFM family of transition metal phosphorus trichalcogenides by pointing out the mechanisms which govern the benchmark compounds, thus extending the fundamental knowledge of 2D magnetism.

## Supporting Information Available

Exchange interactions calculated within DFT + *U* and (II) Wannier analysis (PDF)

## Footnotes

a The second neighbor coupling is very small and we leave it out from the analysis.

b Note that the finite Coulomb repulsion cost on sulfur just renormalizes the (anyway quite hard to estimate) charge transfer energy.
