# Supplementary material for: Limited Ferromagnetic Interactions in Monolayers of MPS3 (M = Mn and Ni)
Source: J Phys Chem C Nanomater Interfaces. 2022 Apr 12;126(15):6791–802. doi: 10.1021/acs.jpcc.2c00646 (PMC9037203; doi:10.1021/acs.jpcc.2c00646)
Supplement: Supplementary file 1 — jp2c00646_si_001.pdf [file jp2c00646_si_001.pdf]

Supporting Information for Publication: ”Limited Ferromagnetic Interactions in Monolayers of MPS_3 (M=Mn, Ni)”

Carmine Autieri and Giuseppe Cuono
International Research Centre Magtop, Institute of Physics, Polish Academy of Sciences, Aleja Lotników 32/46, PL-02668 Warsaw, Poland

Canio Noce
Dipartimento di Fisica “E.R. Caianiello”, Università degli Studi di Salerno, I-84084 Fisciano (SA), Italy

Milosz Rybak
Department of Semiconductor Materials Engineering, Faculty of Fundamental Problems of Technology, Wrocław University of Science and Technology, Wybrzeże Wyspiańskiego 27, PL-50370 Wrocław, Poland

Kamila M. Kotur, Cliò Efthimia Agrapidis, and Krzysztof Wohlfeld
Faculty of Physics, University of Warsaw, Pasteura 5, PL-02093 Warsaw, Poland

Magdalena Birowska*
University of Warsaw, Faculty of Physics, 00-092 Warsaw, Pasteura 5, Poland

(Dated: March 30, 2022)

CONTENTS

I. Exchange interactions calculated within DFT+U S1

II. Wannier analysis S2

References S3

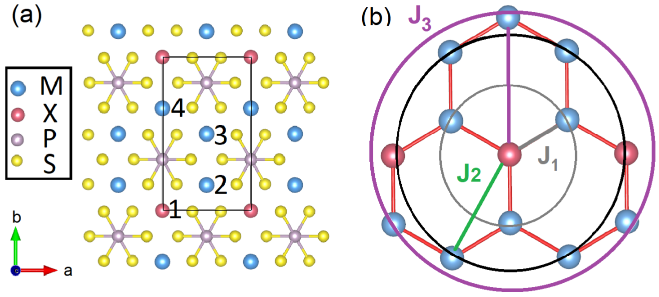

FIG. S1. (a) Rectangular planar cell denoted in black (smallest possible supercell for the impurity concentration of 25%). (b) Schematic picture of the first (J_1, grey circle), second (J_2, green circle) and third (J_3, violet circle) nearest neighbour exchange couplings. Note that the spin arrangements for AFM-N, AFM-z, AFM-s are 1234=↑↓↑↓, ↑↑↓↓, ↑↓↓↑, respectively.

* Magdalena.Birowska@fuw.edu.pl

I. EXCHANGE INTERACTIONS CALCULATED WITHIN DFT+U

To examine the 2D magnetic structure of the employed alloys, we consider the classical spin Hamiltonian on honeycomb lattice, including exchange interactions between nearest-neighbours (1NN), second nearest-neighbours (2NN), and third nearest-neighbours (3NN) between the metal atoms of the host (denoted as J_i^M with i=1,2,3 as the number of neighbour) and between the host and dopant atoms (marked as J_i^{XM} with i=1,2,3). In order to do it, we have chosen the smallest possible supercell (planar rectangular cell, see Fig. S1) for the employed concentration. We insert the notation XMMM, in which the order indicates the first, the second, the third and the fourth atoms in the position represented in Fig. S1. For the simplicity we skipped the letters in notation where applicable and we considered just the spins, e.g. $E_{\downarrow\uparrow\downarrow\downarrow}$ denoted the X atom with spin down, the M atom of the host on 2nd 3rd 4th positions with spin up, down, down components, respectively.

To extract the J_i constants, we fix the lattice constants to that of the most energetically favorable spin arrangement of the host (AFM-Néel for $MnPS_3$ or AFM zig-zag for $NiPS_3$) and calculate the the energies for different spin configurations. We derive the following equations

for all non-equivalent magnetic configurations:

$$\begin{aligned}
E_{\uparrow\uparrow\uparrow\uparrow} &= E_0 + (3J_1^M + 7J_2^M + 3J_3^M)|\vec{S_M}|^2 + J_2^X|\vec{S_X}|^2 \\
&\quad + (3J_1^{XM} + 4J_2^{XM} + 3J_3^{XM})|\vec{S_X}||\vec{S_M}| \\
E_{\downarrow\uparrow\uparrow\uparrow} &= E_0 + (3J_1^M + 7J_2^M - 3J_3^M)|\vec{S_M}|^2 + J_2^X|\vec{S_X}|^2 \\
&\quad + (-3J_1^{XM} - 4J_2^{XM} + 3J_3^{XM})|\vec{S_X}||\vec{S_M}| \\
E_{\downarrow\uparrow\downarrow\uparrow} &= E_0 + (-3J_1^M + 7J_2^M - 3J_3^M)|\vec{S_M}|^2 + J_2^X|\vec{S_X}|^2 \\
&\quad + (-3J_1^{XM} + 4J_2^{XM} - 3J_3^{XM})|\vec{S_X}||\vec{S_M}| \\
E_{\downarrow\uparrow\downarrow\downarrow} &= E_0 + (-J_1^M - J_2^M + 3J_3^M)|\vec{S_M}|^2 + J_2^X|\vec{S_X}|^2 \\
&\quad + (J_1^{XM} + 4J_2^{XM} - 3J_3^{XM})|\vec{S_X}||\vec{S_M}|
\end{aligned}$$

$$\begin{aligned}
E_{\uparrow\uparrow\uparrow\downarrow} &= E_0 + (J_1^M + J_2^M - 3J_3^M)|\vec{S_M}|^2 + J_2^X|\vec{S_X}|^2 \\
&\quad + (-J_1^{XM} + 2J_2^{XM} + 3J_3^{XM})|\vec{S_X}||\vec{S_M}| \\
E_{\downarrow\uparrow\uparrow\downarrow} &= E_0 + (-J_2^M - 3J_3^M)|\vec{S_M}|^2 + J_2^X|\vec{S_X}|^2 \\
&\quad + (-4J_2^{XM} - 3J_3^{XM})|\vec{S_X}||\vec{S_M}| \\
E_{\uparrow\uparrow\downarrow\uparrow} &= E_0 + (-3J_1^M + 7J_2^M - 3J_3^M)|\vec{S_M}|^2 + J_2^X|\vec{S_X}|^2 \\
&\quad + (3J_1^{XM} - 4J_2^{XM} + 3J_3^{XM})|\vec{S_X}||\vec{S_M}| \\
E_{\uparrow\uparrow\downarrow\downarrow} &= E_0 + (-J_1^M - J_2^M + 3J_3^M)|\vec{S_M}|^2 + J_2^X|\vec{S_X}|^2 \\
&\quad + (-J_1^{XM} - 4J_2^{XM} + 3J_3^{XM})|\vec{S_X}||\vec{S_M}|,
\end{aligned} \tag{S1}$$

where $|\vec{S_X}|$ and $|\vec{S_M}|$ are the average spin magnetic moments of the atoms X and M, respectively. We assume the J_i^M are calculated from the host compounds (pure phases) and we compared with the similarly reported for the pure phases [1]. Then, for each of the alloy we have chosen its four lowest energies out of eight presented above, in order to extract the J_i^{XM} and J_2^X mixed exchange couplings.

II. WANNIER ANALYSIS

To calculate the competition between the magnetic couplings in $NiPS_3$ described in the formulas (5) and (6) of the main text, we have changed the strategy since we had to include the hybridization between the p-orbitals and antisymmetric d-orbitals (e_g orbitals if we neglect the trigonal distortions).

In the simplified ionic picture of the $NiPS_3$, we have Ni^{+2}, P^{+4} and S^{-2}. At the first-order of hybridization, we have the *pd* hybridization between the 3*d*-electrons of the Ni and 3*p*-states of the S atoms and the bonding-antibonding between the 3*s*-orbital of the P atoms. At the second-order of the hybridization, we have an hybridization between the 3s-orbitals of the P and 3p-orbitals of the S forming the cluster PS_3 having oxidation state -2 and sp^2-orbitals. However, for our purposes, we can stop at the first order of hybridization, e.g., integrating out the *s*-orbital of the P atoms and mapping the low energy band structure to a model Hamiltonian considering only the antisymmetric Ni-3*d* and S-3*p* orbitals. We calculated the band structure of the nonmagnetic phase of the $NiPS_3$ and we plot it in Fig. S2. The antisymmetric Ni-3*d* bands are above the Fermi energy but they will

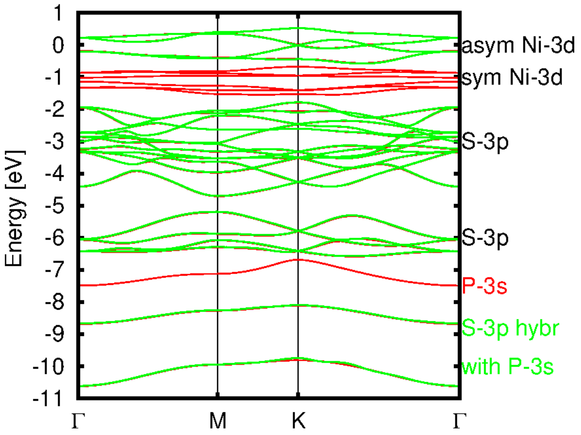

FIG. S2. Band structure of the nonmagnetic phase of the $NiPS_3$. Calculated GGA bands (red lines) and Wannier functions associated with the antisymmetric Ni-3*d* and S-3*p* orbitals (green lines). We labeled the bands with their main orbital contribution. The S-3p bands range from -2 to -11 eV with the two lowest bands that are strongly hybridized with the P-3s bands. The asym and sym Ni-3d states are the even and odd states, respectively.

be at the Fermi level once magnetism is turned on. The bonding state of the P-3*s* is at -7.5 eV from the Fermi level in Fig. S2 while the antibonding is above 2 eV (not shown). Among the occupied bands there are all the S-3*p* bands and one P-3*s* band. The strength of the sp^2 hybridization is made clear from the similar shape of the bands between -11 and -7 eV in Fig. S2. We are able to map part of the band structure on a pd tight binding model, the electronic structure of the pd tight binding model is shown in green in Fig. S2 and it is in good agreement with the DFT band structure shown in red. The numerical values of the relevant hopping parameters and difference in the energies on site Δ are reported in the main text.

[1] N. Sivadas, M. W. Daniels, R. H. Swendsen, S. Okamoto, and D. Xiao, Magnetic ground state of semiconducting transition-metal trichalcogenide monolayers, Phys. Rev. B **91**, 235425 (2015).
